# Orthotopic Models Using New, Murine Lung Adenocarcinoma Cell Lines Simulate Human Non-Small Cell Lung Cancer Treated with Immunotherapy

**DOI:** 10.3390/cells13131120

**Published:** 2024-06-28

**Authors:** Eric P. Knott, Emily Y. Kim, Edison Q. Kim, Rochelle Freire, Justin A. Medina, Yujie Wang, Cheng-Bang Chen, Chunjing Wu, Medhi Wangpaichitr, Jose R. Conejo-Garcia, Diane C. Lim

**Affiliations:** 1 Research Services, Miami VA Healthcare System, Miami, FL 33125, USA; e.knott@med.miami.edu (E.P.K.); emily.kim1@va.gov (E.Y.K.); edison.q.kim@gmail.com (E.Q.K.); chunjing.wu@va.gov (C.W.); mwangpaichitr@med.miami.edu (M.W.); 2 Division of Pulmonary & Critical Care Medicine, Miami VA Healthcare System, Miami, FL 33125, USA; 3 South Florida Veterans Affairs Foundation for Research and Education, Miami, FL 33125, USA; 4 Department of Pathology, University of Miami Miller School of Medicine, Miami, FL 33136, USA; roc.freire@gmail.com; 5 Department of Medicine, University of Miami, Miami, FL 33136, USA; justinamedina1993@gmail.com; 6 Department of Industrial and Systems Engineering, University of Miami, Coral Gables, FL 33146, USA; yxw509@miami.edu (Y.W.); cxc1920@miami.edu (C.-B.C.); 7 Department of Surgery, Cardiothoracic Surgery, University of Miami, Miami, FL 33136, USA; 8 Department of Integrative Immunobiology, Duke University School of Medicine, Durham, NC 27710, USA; jose.conejo-garcia@duke.edu; 9 Division of Pulmonary/Critical Care/Sleep, University of Miami, Miami, FL 33136, USA; 10 Division of Sleep Medicine, Miami VA Healthcare System, Miami, FL 33125, USA

**Keywords:** lung cancer, non-small cell lung cancer, NSCLC, lung adenocarcinoma, mouse models, orthotopic murine lung cancer models, immunotherapy, monoclonal cell lines, Lewis lung carcinoma, surface protein C, club cell

## Abstract

Understanding tumor–host immune interactions and the mechanisms of lung cancer response to immunotherapy is crucial. Current preclinical models used to study this often fall short of capturing the complexities of human lung cancer and lead to inconclusive results. To bridge the gap, we introduce two new murine monoclonal lung cancer cell lines for use in immunocompetent orthotopic models. We demonstrate how our cell lines exhibit immunohistochemical protein expression (TTF-1, NapA, PD-L1) and common driver mutations (KRAS, p53, and p110α) seen in human lung adenocarcinoma patients, and how our orthotopic models respond to combination immunotherapy in vivo in a way that closely mirrors current clinical outcomes. These new lung adenocarcinoma cell lines provide an invaluable, clinically relevant platform for investigating the intricate dynamics between tumor and the immune system, and thus potentially contributes to a deeper understanding of immunotherapeutic approaches to lung cancer treatment.

## 1. Introduction

Over the past century, remarkable scientific advancements have paved the way for the development of drug therapies for lung cancer. From platinum-based chemotherapy to targeted gene therapy, and most recently, immunotherapy, these discoveries have relied on the use of syngeneic preclinical models. The development of immortal murine lung cancer cells started in the 1950s and since then, dozens of cell lines were developed from spontaneous murine lung tumors using diverse methodologies. Four cell lines are notable for their consistent ability to produce orthotopic tumors with acceptable initiation times: the Lewis lung carcinoma (LLC) cell line established in 1951 [1], the Madison (MAD109) cell line established in 1964 [2], the KLN-205 cell line established in 1971 [3,4], and the Carcinosarcoma Metastasis Tumor (CMT) 64/167 cell lines established in 1976 [1,5,6]. However, as our understanding of human lung cancer has increased, the utility of these and other preclinical models has been called into question due to challenges in translating results from mice to clinical trials [7,8]. Over time, differences between these murine lung cancer cell lines and known human lung cancers have been implicated, including variations in histologic growth patterns, inconsistent protein markers and oncogenic driver mutations [9].

Across the globe, cancer continues to be a major burden in disability-adjusted life years, mortality and years of life lost, second only to cardiovascular disease [10]. Unfortunately, tracheal, bronchus and lung (TBL) cancers continue to have the highest cancer related mortality. While cigarette smoking remains a major risk factor developing TBL cancers [11], there is increasing evidence that previous pulmonary tuberculosis [12], fine particulate matter air pollution [13] and unhealthy lifestyle behaviors [14] increase risk of lung cancer specific mortality. While the evidence of electronic cigarettes on lung cancer is not yet available [15], there is increasing evidence that they elicit an inflammatory response [16] and epigenetic effects [17] that are predictive of carcinogenesis. Historically, lung cancer is divided into two main histological subtypes, non-small cell lung cancer (NSCLC) and small cell lung cancer (SCLC). NSCLC is further divided into two main subtypes, adenocarcinoma and squamous cell carcinoma (SCC), based on tumor cell morphology and growth patterns [18,19]. Histologic subtyping of NSCLC remains important today, as it tends to correlate with treatment response and clinical outcomes [20]. For example, vinorelbine therapy improved survival in patients with SCC histology [21] while bevacizumab and pemetrexed are contraindicated for SCC due to the risk of severe pulmonary hemorrhage [22] and treatment inferiority [23].

In 2011, the International Association for the Study of Lung Cancer, together with the American Thoracic Society and European Respiratory Society, published a revised classification system for NSCLC placing greater emphasis on immunohistochemical protein expression markers over histologic evaluation [19]. Current clinical algorithms for the diagnosis of human lung adenocarcinoma typically require positivity of stains for thyroid transcription factor 1 (TTF-1) and aspartic proteinase Napsin A (NapA), and negativity of stains for either p40 or p63 [24]. TTF-1, also known as NK2 homeobox 1 (NKX2-1), is a member of the homeodomain-containing transcription factor family, and under non-malignant conditions, activates the expression of select genes in the thyroid, lung, and brain [25]. In lung adenocarcinoma, some postulate that TTF-1 seems to promote survival and growth of cancer, but paradoxically, inhibit invasion, metastasis, and progression [26]. Despite a large meta-analysis linking TTF-1 overexpression in human lung adenocarcinoma with improved survival, the data is largely conflicting [27]. NapA is an enzyme involved in the maturation of pro-surfactant protein B (SPB) in type II pneumocytes [28], and under non-malignant conditions, may have a role in phagocytosis by alveolar macrophages [29]. In cases of lung adenocarcinoma, under malignant conditions NapA may be regulated by TTF-1, where low levels of NapA lead to TGF-β induced neoplastic cell proliferation [30]. Lastly, the presence of p40, a variant isoform of the p63 protein, is highly specific for lung SCC [31,32,33,34]. Based on current knowledge, no existing murine lung cancer cell lines accurately mirror the immunohistochemistry protein expression patterns observed in human lung adenocarcinoma.

In addition to histological evaluation and immunohistochemical protein expression, molecular analyses for oncogenic driver mutations have emerged as important diagnostic tools in the field of lung cancer. First, they aid in the diagnosis and classification of lung cancer subtypes, as there are distinct genetic profiles that differentiate lung adenocarcinoma and lung SCC [35]. For example, *KRAS* and *EGFR* mutations frequently occur in adenocarcinoma, while SCC has a much lower prevalence of these mutations [35]. Second, molecular testing can assist with identifying therapeutic gene targets. In the case of adenocarcinoma, potential targets include *KRAS*, *EGFR*, *ALK*, *ERBB2*, and/or *BRAF* [36,37,38]. These molecular mutations inform treatment decisions and enable personalized therapies. Third, rare somatic mutations can provide prognostic information [35]. For example, *PIK3CA* mutations are present in 1–2% of all lung adenocarcinomas [39] and their constitutive activation in downstream pathways has been associated with drug resistance [40,41]. While p53 mutations are prevalent in ~70% of adenocarcinomas [42,43] and nearly all squamous cell carcinomas, testing for this mutation is not typically included in the diagnostic algorithm for differentiating adenocarcinoma and SCC, however it can have prognostic significance [44].

Following these methods to diagnose NSCLC, pathologists will routinely quantify specific tumor markers to help inform anti-cancer drug treatment. In recent years, immunotherapy has emerged as one of the most significant scientific breakthroughs for the treatment of metastatic NSCLC. Initially, immunotherapy demonstrated a significant improvement in overall survival (OS) of patients with advanced NSCLC. However, subsequent large-scale trials reveal that only ~20% of advanced NSCLC patients experienced a therapeutic benefit [45]. Thus, the pre-treatment calculation of the percentage of biopsy tumor cells expressing the Programmed Death-Ligand 1 (PD-L1) protein, termed the tumor proportion score (TPS) for PD-L1, emerged as a clinical tool to predict efficacy of anti-programmed cell death protein 1 (PD-1) or anti-PD-L1 immunotherapy [46,47,48]. However, despite known positive expression of PD-L1 by each of the four cell lines previously discussed, each displays varying degrees of in vivo primary resistance to immunotherapy [49,50,51], for reasons that remain unclear.

In summary, while preclinical models are critical to understanding tumor–host immune interaction, their utility is dependent on their ability to recapitulate human lung cancer. Without accurate representation of human lung cancer, investigations into the mechanisms underlying primary and secondary resistance to immunotherapy using preclinical models may yield inconclusive or non-translatable results. To address these critical issues, we developed new murine lung cancer cell lines with the aim of fulfilling three limitations of existing cell lines: (1) when injected orthotopically, tumors have a histology consistent with the most common lung cancer, adenocarcinoma; (2) cells express immunohistochemical protein expression markers that are clinically relevant and characteristic of lung adenocarcinoma; (3) cells express immunogenic antigens in vitro and simulate in vivo, the clinical response and resistance patterns observed in patients undergoing immunotherapy.

## 2. Methods

The animal study protocol was reviewed and approved by the institutional animal care use committees (IACUC) of the Perelman School of Medicine at the University of Pennsylvania and the Miami Veterans Health Administration.

### 2.1. Materials/Data Availability

There are no restrictions on the availability of our cell lines. At the time of submission, the process of cell banking had been initiated with Kerafast, Inc. (Boston, MA, USA) but is not yet completed. If the lines remain unavailable via Kerafast, for any reason, we will be glad to share our cells following receipt of a materials transfer agreement with the requestor.

### 2.2. Cell-Type Specific Cre-Recombinant Adenoviral Vectors

Two cell-specific Cre-recombinant viral vectors were purchased from the University of Iowa Viral Vector Core. These viruses were developed by Berns et al. [52,53] and employ the Cre-loxP system with a DNA promoter sequence of choice that serves to deliver Cre recombinase only to the targeted Cre-expressing cells. The Ad5SPCCre virus targets cells with Surfactant Protein C (SPC), a 21 kDA integral membrane precursor protein that is critical to the properties of pulmonary surfactant and is only synthesized by the alveolar type 2 (AT2) cell. Similarly, the Ad5CC10Cre virus targets cells with Clara Cell Protein 10 (CC10), a 10-kDA non-glycoprotein that is part of the secretoglobulin family and may have a role as an immunomodulator. In the lung, CC10 is synthesized by the Club cells, a type of bronchiolar epithelial cell that secretes mucin-products important in protecting the bronchiole from inhaled microorganisms.

### 2.3. Genetically Engineered Mouse (GEM) Model of Lung Cancer

Generation of a KRAS^G12D+/−^ p53^fl/fl^ myristoylated-p110α^fl/fl^-ROSA-gfp was developed by Fiering et al. [54,55,56] and has been previously described. Briefly, transgenic Kras^G12D+/−^ and p53^fl/fl^ mutant mice were obtained from the National Cancer Institute Mouse Models of Human Cancers Consortium, were brought to a full C57BL/6 background [57], and bred with C57BL/6 myristoylated-p110α^fl/fl^-ROSA-gfp mice. The conditional triple mutant mice produced were genotyped using PCR as previously described [55], to confirm heterozygosity for Kras^G12D^ and homozygosity for p53^fl/fl^ and myristoylated-p110α^fl/fl^-ROSA-gfp [56]. Administration of Cre-virus to activate oncogenic driver mutations is performed by direct intrapulmonary injection under general anesthesia. Further details regarding survival surgery for intrapulmonary administration of Cre virus are detailed below.

### 2.4. Mouse Husbandry

Non-breeding animals were housed in sex-matched groups of five. Living conditions were strictly monitored and remained consistent throughout the experimental period. The vivarium maintained a temperature of 72 °F, with humidity levels ranging from 40% to 60%. Mice were housed in cages equipped with 1/8” corncob bedding and provided with a Bio-Serv Mouse Igloo for enrichment. They were provided a diet of water and rodent diet pellets. The pellets consisted of ground wheat, ground corn, and dehulled soybean meal, formulated to provide 18% protein, 6% fat, and 44% carbohydrates per serving, free from animal protein or fish meal. The vivarium staff changed cages weekly and visually inspected mice daily. Any mice displaying signs of distress, such as improper grooming or severe weight loss (>15% total body weight) were promptly harvested and/or euthanized based on their experimental group assignments.

For breeding, one male, triple transgenic KRAS^G12D+/−^ mouse was housed with two female, triple transgenic KRAS^G12D−/−^ mice. Subsequently, after litters were born, they were transferred to hamster-sized cages to prevent overcrowding. Rat-sized cages were maintained with identical bedding, food, and water sources. Pregnant females were additionally provided with sunflower seeds just prior to giving birth to promote lactation and breastfeeding.

### 2.5. In Vivo Checkpoint Inhibitor Participants/Orthotopic Modeling

A total of ninety-six wildtype C57BL/6 mice, aged 12 to 13 weeks, were randomly assigned to one of three cohorts and orthotopically injected with LLC, SmKPP.1, or CmKPP.1. Micro Computed Tomography (µCT) scans were generally performed one week after injection, and tumor volumes were calculated for each mouse. Mean tumor volumes were calculated using an equation for the volume of an oval. Initially, different concentrations of clone cells were evaluated. Then when evaluating response to checkpoint inhibitors, within each cohort, mice were randomly selected and allocated using an approximation of 2:1 system (2 control for every 1 treated with immunotherapy), and they received weekly intraperitoneal injections of combination anti-PD-1 (BioXCell, InVivoMAb anti-mouse PD-1 (CD279), #BE0033-2, given on Tuesdays) and anti-PD-L1 (BioXCell, InVivoMab anti-mouse PD-L1 (B7-H1), #BE0101, given on Thursdays), at a dose of 10 mg/kg; control mice were injected with sterile PBS (also given on Tuesdays and Thursdays).

### 2.6. Cre Virus Activation and Preparation

Ad5mSPC-Cre [58] (University of Iowa Viral Vector Core, VVC-Berns-1168-HT) and Ad5CC10-Cre [58] (University of Iowa Viral Vector Core, VVC-Berns-1166) virus was precipitated according to instructions. Specifically, we used a 4:34 dilution of 20 mM CaCl_2_:1× Dulbecco’s Modified Eagle’s Medium (DMEM) for 20 min.

### 2.7. Murine Survival Surgery

Mice were anesthetized with inhaled isoflurane. Induction of anesthesia was achieved using 5% isoflurane in 100% oxygen administered at a flow rate of 1.5 L/min. After checking for pedal reflexes, mice received subcutaneous (SQ) injections of 0.03 mg/kg buprenorphine XR and 5 mg/kg of meloxicam, and maintenance of anesthesia was achieved using 2–3% isoflurane in 100% oxygen administered the same flow rate. Using small animal nail clippers, hind toenails were clipped to prevent self-injury post-operatively [56]. The mouse was positioned such that the left lung was up and was shaved using a 0.5 mm beard shaver. The surgical site was cleaned with 70% isopropyl alcohol then with chlorhexidine and repeated a total of three times. An incision approximately 1 cm in length was made and blunt dissection using scissors was used to visualize the left lung. 10 µL of the activated virus was drawn into a 29G 1/2” insulin syringe and injected in between the 9th and 10th intercostal rib. Tissue adhesive was used to close the incision. Following the procedure, the mice were warmed for two to five minutes under a heat lamp, until they regained consciousness and showed basic activity and functionality before being returned to their original cages. All mice were monitored daily for signs of distress, such as tachypnea or bradypnea, decreased movement, reduced body weight, poor grooming, or wound dehiscence. For mice with recoverable wound dehiscence within a seven-day period after the procedure, a second dose of subcutaneous buprenorphine 0.5 mg/kg XR with meloxicam 5 mg/kg were administered, and they were separated from cage mates for up to one week to facilitate healing. Except for the injected material, this protocol was performed identically for the formation of orthotopic tumors using clone cells. To evaluate the response to immunotherapy, orthotopic injection was performed by injecting 1.25 × 10^5^/15 µL 1× PBS of either LLC, SmKPP.1 or CmKPP.1.

### 2.8. Computed Tomography

Most mice underwent micro computed tomography (µCT) of the chest one week following survival surgery and typically every two weeks thereafter (there were times when mice could not be scanned due to issues out of our control). At the University of Pennsylvania, we utilized the Molecubes X-cube and at the Miami VA we utilized the Bruker SkyScan 1176 Micro-Computed Tomography scanner. For image acquisition, mice were anesthetized using continuously inhaled isoflurane at 1.5 L/min administered via nosecone. To minimize radiation exposure and improve image quality, respiratory gating was employed in real time by way of computer software analysis. The source voltage and current for the X-cube: 50 kV, 440 µA, 480 exposures at 125 ms/exposure for a total radiation dosing for our animals was 48 mGy per scan; images were reconstructed to 100 μm. The source voltage and current for the SkyScan 1176: 50 kV and 400 µA, and radiation was filtered through a 0.5 mm Aluminum plate; the axial step and shoot acquisition modality resulted in an exposure time was 71 ms per image and each scan acquired approximately 200 images to achieve a final resolution of approximately 34 um per pixel; a fixed source-to-object distance of 121.7 mm and assuming the average adult mouse has a thoracic diameter of 25 mm, total radiation dosing for our animals was 31.4 mGy per scan. Images acquired from both scanners were analyzed using ITK Snap version 3.8.0 (http://www.itksnap.org/), a free, open-source, multi-platform software application used to segment structures in 3D and 4D biomedical images [59]. The software allows users to co-localize areas of interest in three planes: axial, sagittal, and coronal. 

### 2.9. Mouse Euthanasia and Harvesting

Prior to dissection and tissue collection, all mice were deeply sedated to ensure humane and ethical practices. During the dissection process, careful assessment of local and distant metastasis was conducted, and lymph node size in the mediastinum, mesenteric/paraspinal, and axillary regions were classified. Tissue designated for cell sorting was immediately dissected and placed in a 24 well plate filled with fresh 1× PBS. Tissue intended for H&E or immunofluorescence (IF) were collected only after the mouse was perfused. Perfusion was conducted by administering 30 mL of 0.9% normal saline directly into the right ventricle of the heart using gravity, then 30 mL of 4% formaldehyde (formalin) was administered to fix the tissues for H&E and IF analyses.

### 2.10. Cell Lineage Generation, Cell Sorting, and Cell Culture

The mice selected for clone cell development were chosen from a group of mice being used for a separate project, specifically the evaluation of survival as a function of Cre concentration. Two groups of ten genetically engineered mice (GEM) with the **K**RAS^G12D+/−^/**p**53^fl/fl^/myristoylated-**p**110α^fl/fl^-ROSA-gfp genotype, aged 6 weeks to 8 weeks, were injected; n = 5 males and n = 5 females were injected with Ad5SPCcre and n = 5 males and n = 5 females were injected with Ad5CC10cre. A single mouse was selected for harvest from each group when tumor volume in the left lung measured by uCT scan approximated 33% of the left hemithorax; remaining mice continue to be a part of the survival study. This size was intentionally chosen to maximize the number of viable cells for growth while minimizing the degree of necrotic tissue. Specifically, **S**mKPP.1 was generated by injecting 10^6^ Ad5**S**PCCre virus into the left lung of a male GEM; 12 weeks after the injection the mouse was harvested, and the entire left lung underwent single cell suspension. Similarly, **C**mKPP.1 was generated by injecting 10^5^ Ad5**C**C10Cre virus into the left lung; 13 weeks after injection the mouse was harvested, and the entire left lung underwent single cell suspension.

Single cell suspension of the left lung of each mouse was performed by mincing tissue and suspending the tissue in 5 mL of DMEM containing 2 μg/mL Collagenase + 0.001% DNase I and incubated for 15 min at 37 °C to allow for enzymatic digestion. The solution was filtered dropwise through a 40 µm mesh (Falcon, Corning Inc., Corning, NY, USA) into a 50 mL conical tube, and 10 mL of prewarmed RPMI was added to wash the filter. The final mixture was then centrifuged at 1000× *g* for 5 min at 4 °C, and the supernatant was discarded. The resulting pellet was resuspended in 5 mL of red blood cell lysis buffer and incubated for 2 min at room temperature. The suspension was then quenched by adding 15 mL of Fluorescence Activated Cell-Sorting (FACS) buffer and spun at 1000× *g* for 5 min at 4 °C.

The supernatant was discarded, and the pellet was resuspended in 1 mL of FACS buffer and gently agitated. Ten microliters (µL) were set aside for cell counting, and four 200 µL aliquots were set aside to serve as positive controls for fluorochromes, including CD45 and CD31, Zombie Yellow, and GFP. An antibody cocktail was prepared by adding CD45, CD31, and Zombie Yellow at a dilution of 1:100 to DPBS. For every million live cells counted, 1 µL of the antibody cocktail was added to the cell suspension and the mixture was incubated in the dark at room temperature for 20 min. After incubation, 5 mL of FACS buffer was added to the suspension as another quench, and the cells were centrifuged at 1000× *g* for 5 min at 4 °C. The supernatant was discarded, and the pellet was resuspended in sufficient FACS buffer to achieve a final concentration target range of 5 × 10^6^ to 10 × 10^6^ live cells/mL.

After cellular staining was complete, six 96-well plates were prepped with 200 µL of prewarmed RPMI 1640 (1×) medium, supplemented with L-Glutamine (2 mM), 25 mM HEPES, 10% fetal bovine serum, 100 IU/mL Penicillin, and 100 μg/mL Streptomycin. For each tumor type (i.e., each mouse harvested or each respective viral injection), both the control solutions and the stained cells were processed through a single-cell sorter such that endothelial, immune, and dead cells were discarded, while gfp-positive cells could be plated individually into the single wells of a 96-well plate. The 96-well plates were maintained in a 5% CO_2_ incubator at 37 °C and visual inspection confirmed a single cell in each well. Cultures were monitored daily, and media was added as needed. When the culture reached 80% confluence, it was trypsinized, counted and replated. In this way, we scaled up from one well of a single 96-well plate to four wells of 96-well plate, and then from four wells to eight wells, and so on until graduating to a T-25 and finally T-75 flasks.

### 2.11. Cell Storage and Orthotopic Preparation

Upon reaching 80–90% confluency, cultured cells were processed under a sterile biosafety cabinet. The media was aspirated, and the adherent cells were washed with sterile 1× PBS (without Calcium Chloride or Magnesium Chloride). Subsequently, the 1× PBS was aspirated and discarded. Pre-warmed 0.25% trypsin (2.21 mM EDTA, 1× [−] sodium bicarbonate) was added and allowed to rest for 1–2 min, or until approximately 90% of the cells detached. The trypsin was then neutralized with the addition of fresh media (approximately three times the volume of trypsin in the flask). The cell suspension was then centrifuged at 200× *g* for 5 min at room temperature, and the supernatant was aspirated and discarded. The cell pellet was resuspended in 2 mL of sterile, pre-warmed RPMI. Gentle agitation with a 1 mL pipette was employed to ensure homogeneity. A 100 µL aliquot was set aside for cell viability assessment and counting using the TC20 Automated Cell Counter. The remaining volume of cells underwent a second centrifugation for 5 min at 200× *g* at room temperature.

For orthotopic modeling experiments, the viable cell count was used to resuspend the cell pellet in an exact volume of PBS, achieving desired concentrations of cells per 15 µL of 1× PBS. Subsequently, 15 μL aliquots were transferred to sterile Eppendorf tubes and kept on ice until ready for injection. For cell storage, the viable cell count was used to calculate the appropriate volume of freezing media (90% deactivated fetal bovine serum and 10% DMSO) for resuspension, resulting in a final concentration of 2M cells/mL. Aliquots (1 mL each) were transferred to storage vials, half-submerged in 70% ethanol within a Mr. Frosty storage container, and gradually chilled to −80 °C. After twenty-four hours, the vials were individually transferred to liquid nitrogen vapor for long-term storage. For orthotopic experiments, the clone cells were thawed from liquid nitrogen, passaged twice, and allowed to grow in culture until reaching 80–90% confluency.

### 2.12. Tissue Preservation and Preparation

Following harvest and tissue fixation in formalin overnight, tissue was then immersed in 30% sucrose for forty-eight hours before being embedded in optimal cutting temperature compound (OCT) using a Peel-A-Way disposable histology mold. Subsequently, a cryostat was used to obtain 10 μm-thick tissue sections. To maintain the natural architecture of the murine lung during cryoslicing, we applied an adherent cryofilm (Section-lab, Hiroshima, Japan) [60] directly to the front of the tissue fixed embedded in OCT and adhered to the pedestal. After slicing, the cryofilm with the tissue slice, was securely affixed to a microscope cover slip using a small drop of CytoSeal 60 on the non-adherent side of the film. To ensure proper sealing, any excess CytoSeal 60 around the edges was carefully removed. The cover slip with the newly affixed cryofilm and tissue, was allowed to dry overnight at 4 °C. The cover slip with cryofilm and tissue could be immediately used for immunohistochemistry (IHC) or immunofluorescence (IF) assays, or temporarily stored at −20 °C.

Tissue slices undergoing histopathologic analysis or immunofluorescence were rinsed with 1× PBS for 1–2 min ×3 to eliminate any residual OCT from the adherent side of the cryofilm, then air-dried for 1 h at room temperature. Considering the delicate nature of the tissue, we adopted a stainless-steel base mold (32 mm × 25 mm × 12 mm) as an alternative to traditional slide staining racks or dipping systems. For each step of the protocol, the cover slip with the tissue was placed inside the stainless-steel base mold and staining solution was added dropwise to the mold until the tissue was entirely immersed (approximately 400 µL) then placed into a humidifier, onto a rocker, at room temperature.

### 2.13. Histopathology and Immunofluorescence

For immunostaining, cover slip with the tissue on cryofilm was first permeabilized with a solution of 0.4% Triton/PBS for 20 min, followed by two rinses with 1× PBS for about 1–2 min each. We then blocked with 4% Normal Donkey Serum in 1× PBS for two hours, followed by two rinses with 1× PBS. Primary antibody concentrations were optimized in a solution of 1% bovine serum albumin and incubated at room temperature overnight (~16 h). The following morning, tissue was rinsed with 1× PBS for 15 min ×2 then incubated with secondary antibodies for two hours. Last, tissue was rinsed with 1× PBS for 15 min ×2 then incubated with DAPI (Biolegend, San Diego, CA, USA, #422801) for 10 min and rinsed again with 1× PBS for 15 min ×2. The cover slip is mounted onto a microscope slide and stored in an opaque slide case to avoid UV light exposure. Primary antibodies used: CD31 (R&D, Minneapolis, MN, USA, #AF3628, goat), NapA (ThermoFisher, Waltham, MA, USA, #PA5-50329, rabbit, polyclonal), PDL1/CD274 (ABclonal, Woburn, MA, USA, #A1645, rabbit, polyclonal); TTF-1 (Sigma-Aldrich, St. Louis, MO, USA, #07-601, rabbit, polyclonal). Secondary antibodies used: Donkey anti-goat, AF-488 (Invitrogen, Carlsbad, CA, USA, #A-11055) and Donkey anti-rabbit, AF-594 (Invitrogen, #R37119). All images in Figure 3 were obtained at 20× using the same settings: black and white balances, and automatic exposure, which resulted in exposure times of 1 s for (A.1), 0.83 s for (A.2), 0.1 s for (A.3), 0.1 s for (B.1), 0.07 s for (B.2), 0.1 s for (B.3), 0.04 s for (C.1), 0.01 s for (C.2), and 0.2 s for (C.3). A pathologist (RF) reviewed all slides, and digital images were captured using an inverted fluorescence phase contrast microscope.

**Figure 1:**
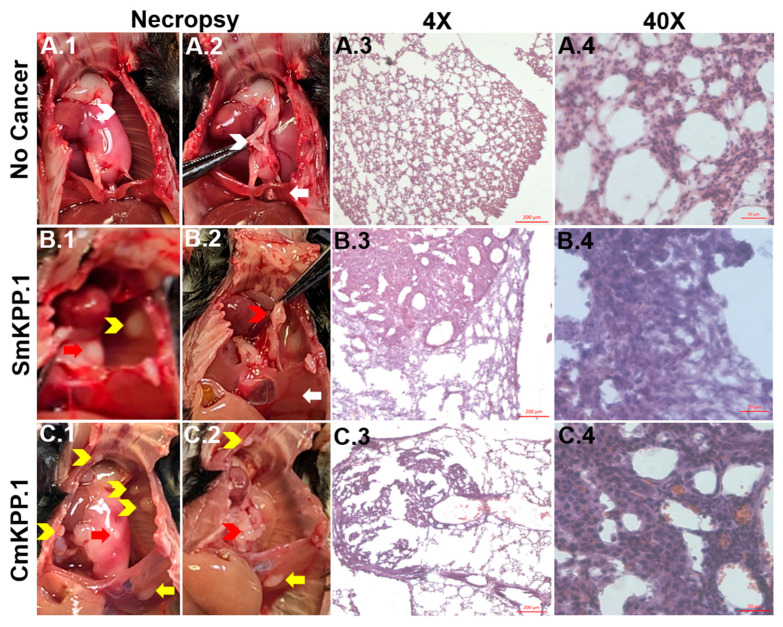
**Visual inspection at necropsy and H&E staining of normal, healthy murine lung tissue and orthotopic tumors generated with monoclonal SmKPP.1 or CmKPP.1 cells.** At necropsy, compared to a mouse with no cancer (**A.1**,**A.2**)), visual confirmation can be made of primary tumor (left lung, (**B.1**,**C.1**), red arrows), metastases to ribs ((**B.1**,**C.1**), yellow chevrons), enlarged mediastinal lymph nodes ((**B.2**,**C.2**), red chevrons) and metastases to the left hemidiaphragm ((**C.1**,**C.2**), yellow arrow). H&E Images at 4× reveal that orthotopic tumors generated by SmKPP.1 (**B.3**) and CmKPP.1 (**C.3**) consist of dense sheets of cells surrounded by healthy lung interstitium and alveoli, which normally appears as a delicate, thin, lacy network of cells (**A.3**). H&E at 40× reveals that both SmKPP.1 (**B.4**) and CmKPP.1 (**C.4**) tumors have abundant cytoplasm and have predominantly vesicular nuclei with notable nucleoli compared to normal healthy lung tissue (**A.4**). All images are 10 μm-thick tissue sections. Scale bars: 200 µm for 4× and 20 µm for 40×.

Tissue stained with Hematoxylin and Eosin were processed following the manufacturer’s instructions (Vector Laboratories, Newark, CA, USA) with slight modifications. First, the cover slip with tissue was incubated with Hematoxylin for 5 min followed by two rinses with distilled water for 15 s each. To enhance contrast and facilitate nuclear staining, tissue was incubated in Bluing Reagent for 15 s followed by two rinses with distilled water for 15 s each; this optimized the visualization of cellular nuclei. The tissue was then dehydrated with increasing concentrations of ethanol (50%, 70%, and 100%), each step lasting 10 s. The tissue was then incubated with Eosin Y for 3 min to stain the cytoplasmic components followed by two rinses with 100% ethanol for 15 s each, then cleared with Xylene and air-dried at room temperature. All images in Figure 1 were obtained using the same settings: white balance and automatic exposure (5 ms for 4× images and 80–90 ms for 40× images); images were cropped for size. Once again, a pathologist (RF) reviewed all slides, and digital images were captured using a light microscope.

### 2.14. Western Blot Analyses

Cultures of each cell line were harvested, and a pellet containing 2 × 10^6^ cells was collected in sterile 2 mL Eppendorf tubes. The pellet was then washed with 1× PBS and centrifuged at room temperature at 200× *g* for 5 min. To ensure protein stability during extraction, 500 µL of protease inhibitor (PI) was added to each sample, vortexed, and centrifuged. The supernatant was discarded, and the pellet was resuspended in 80 µL of Cell Lysis Buffer. The mixture was then sonicated three times at 10-s intervals. For protein quantification, a 2 µL aliquot of the cell lysate was mixed with 598 µL of PBS in a separate Eppendorf tube, creating a dilute protein solution. The remaining cell lysate was supplemented with 40 µL of BME + Laemmli Sample Buffer and heated in a 100 °C heat bath for 10 min to denature the proteins and prepare them for western blot analysis. After cooling to room temperature, the lysate was either used immediately if protein quantification was complete or stored at −80 °C for future use.

For each sample, a 100 µL aliquot of the dilute protein solution was combined with 100 µL of a 1:300 protein assay detection solution (Micro BCA Protein Assay Kit, Thermo Fisher Scientific, Waltham, MA, USA) in two wells of a 96-well plate. Additionally, two control wells were prepared using 100 µL of PBS. The 96-well plate was then incubated at 55 °C for 20 min then cooled at room temperature for 20 min before being analyzed in a plate reader for protein quantification.

Fresh SDS-PAGE gels were made, wells were loaded with either protein lysate (up to 30 µg) or dual color molecular weight marker (Bio-rad, Hercules, CA, USA). The gel was run for 2 h at 100 V using a Mini-Protean Tetra Cell Electrophoresis Chamber. Using a wet transfer technique in 1× transfer buffer, the gel was transferred to an immobilon-P transfer membrane which was previously soaked in methanol for 15–20 min. The transfer cassette was then run for 1.5 h at 100 V Mini Trans-Blot Cell. After the run completed, the transfer membrane was rinsed twice with 1× Tris-buffered saline (TBS) for 5 min on a rocker. The membrane was then submersed in a 5% blotting-grade blocker for 1 h at room temperature then rinsed in 1× TBS for 5 min. Primary antibody concentrations were optimized in a solution of 5% bovine serum albumin and 0.02% sodium azide and incubated in a cold room (4 °C) overnight (~16 h). The following morning, the transfer membranes were washed 3 times in 1× TBS for 15 min each then incubated in secondary antibody (concentration also optimized) for one hour. After another three rounds of 15-min washes in 1× TBS, the membranes were submerged in a 1:1 dilution of SuperSignal West Femto Maximum Sensitivity Substrate (ThermoFischer) for 15 s before image acquisition on the ChemiDoc MP Imaging System (Bio-Rad). Images were obtained using the same settings: white balance and automatic exposure (Bio-rad ChemiDoc MP Imaging System, and acquisition software set to the Chemi-Hi-Sensitivity protocol). Primary antibodies used: Beta-actin (Sigma-Aldrich, #SAB1305554, mouse monoclonal); KRAS^G12D^ (Invitrogen, #MA5-36256, rabbit, recombinant monoclonal); NapA (ThermoFisher, #PA5-50329, rabbit, polyclonal), p40 (ABclonal, #A19616, rabbit, recombinant monoclonal), p53 (Cell Signaling, #30313, rabbit recombinant monoclonal); p110α (Cell Signaling, Danvers, MA, USA, #4249, rabbit, recombinant monoclonal); PDL1/CD274 (ABclonal, #A1645, rabbit, polyclonal); TTF-1 (Sigma-Aldrich, #07-601, rabbit, polyclonal). Secondary antibodies used: Goat anti-rabbit (Promega, #W4011) and Goat anti-mouse (Promega, Madison, WI, USA, #W402B) IgG HRP conjugate.

### 2.15. Statistical Analysis

Prior to conducting the experiments, we performed *a priori* sample size calculations using the G*Power 3 program [61,62]. For the survival analyses comparing mice treated with PBS and immunotherapy, we aimed for a significance level of 5% and a statistical power of 85%. Our goal was to detect an independent mean difference of approximately three weeks in survival between the two groups, with no greater than an intra-cohort standard deviation of 1.5 weeks. Therefore, to achieve at least a small effect, we used effect size (Cohen’s d) of 2 with normality assumption. To ensure balanced allocation of mice to treatment arms, we planned to adopt a 2:1 allocation system. This meant that for every two mice assigned to the control arm (PBS), one mouse would be allocated to the intervention arm (immunotherapy group). Based on this approach, the minimum number of mice required to achieve statistical significance was fourteen (n = 14), with nine (n = 9) being allocated to the control arm and five (n = 5) being allocated to the intervention arm. However, for practical purposes (ease of caging mice together, ease of calculations, etc) we utilized a total sample size of fifteen (n = 15) mice per specific cell type cohort, with ten (n = 10) mice allocated to the control arm and five (n = 5) mice to the experimental arm in each cohort. For both treatment modalities (PBS and immunotherapy), separate one-way analysis of variance (ANOVA) was conducted on starting tumor volumes. Survival results were subjected to statistical analyses using univariate ANOVA, as well as a log-rank test [63].

## 3. Results

### 3.1. New Murine Lung Cancer Cell Lines, SmKPP.1 and CmKPP.1, Are Phenotypically Stable in Culture

We report on two new monoclonal murine lung adenocarcinoma cell lines. **S**mKPP.1 was generated by injecting Ad5**S**PCcre [58] into a **m**ale, Genetically Engineered Mouse (GEM) with the **K**RAS^G12D+/−^/**p**53^fl/fl^/myristoylated-**p**110α^fl/fl^-ROSA-gfp genotype [54]. Similarly, **C**mKPP.1 was derived by injecting Ad5**C**C10cre [58] into a separate, **m**ale GEM (see methods for details). To develop SmKPP.1, 576 single-cell cultures were made from one mouse with an Ad5SPCCre-origin tumor; only one single-cell culture survived to mass storage, translating to a success rate of 0.17%. To progress from single-cell culture to mass storage, SmKPP.1 required nineteen passages over a period of six months with a doubling time over a five-day period of ~18 h. To develop CmKPP.1, 576 single-cell cultures were again made from one mouse with an Ad5CC10Cre-origin tumor; three single-cell cultures independently survived to storage, translating to a success rate of 0.52%. The CmKPP.1 cell line was one of these three and was not mixed with the other three surviving lines; herein we report only on CmKPP.1. To progress from single-cell culture to mass storage, CmKPP.1 required twelve passages over a period of two months with a doubling time over a five-day period of ~12 h. During initial passages before mass storage as well as after mass storage, both cell lines grow stably as adherent, monolayer sheets, with no obvious morphologic or metabolic changes observed; SmKPP.1 and CmKPP.1 cell lines were never mixed. Independent authentication of both cell lines was performed by ATCC using short-tandem-repeat (STR) profiling based on established protocols [64] and the report indicated that the SmKPP.1 and CmKPP.1 cell lines are distinct from each other, and is of murine origin with a 100% match threshold.

### 3.2. New Cell Lines Form Solid Adenocarcinoma Lung Tumors In Vivo

At necropsy, visual inspection of the thorax from a mouse with no cancer (Figure 1(A.1, A.2) can be used to compare and confirm the following: (1) primary tumor (left lung, Figure 1(B.1,C.1), red arrows); (2) metastases to ribs (Figure 1(B.1,C.1), yellow chevrons) compared to normal ribs (Figure 1(A.1), white chevrons); (3) enlarged mediastinal lymph nodes (Figure 1(B.2,C.2), red chevrons), compared to normal mediastinal lymph nodes (Figure 1(A.2), white chevron); (4) metastases to the left hemidiaphragm (Figure 1(C.1,C.2), yellow arrow) compared to normal hemidiaphragm (Figure 1(A.2, B.2), white arrow).

The cellular morphology and behavioral growth patterns of lung tumors injected with either SmKPP.1 or CmKPP.1 is consistent with solid lung adenocarcinoma. Hematoxylin and eosin (H&E) stained orthotopic tumors generated by SmKPP.1 (Figure 1(B.3,B.4)) and CmKPP.1 (Figure 1(C.3,C.4)) clearly distinguishes them from normal (Figure 1(A.3,A.4) lung tissue. At a magnification of 4×, these tumors consist of dense sheets of cells surrounded by healthy lung interstitium and alveoli, which appear as a delicate, thin, lacy network of cells. Further magnification at 40× reveals that both SmKPP.1 (Figure 1(B.4)) and CmKPP.1 (Figure 1(C.4)) tumors exhibit abundant cytoplasm and predominantly vesicular nuclei with notable nucleoli. Additionally, there is an absence of characteristic cellular morphologies associated with squamous cell carcinoma, such as nests of epithelial cells, keratin pearls, or visible intercellular bridges. Although both cell lines exhibit a classic solid histologic growth pattern, CmKPP.1 tumors (Figure 1(C.4)) are more likely to retain some of the surrounding lung architecture as they invade normal lung tissue, occasionally displaying papillary morphology and bearing a vague resemblance to normal lung tissue (Figure 1(A.4)).

### 3.3. SmKPP.1 and CmKPP.1 Cells Resemble Human Lung Adenocarcinoma Gene Products and Protein Expression

**Figure 2:**
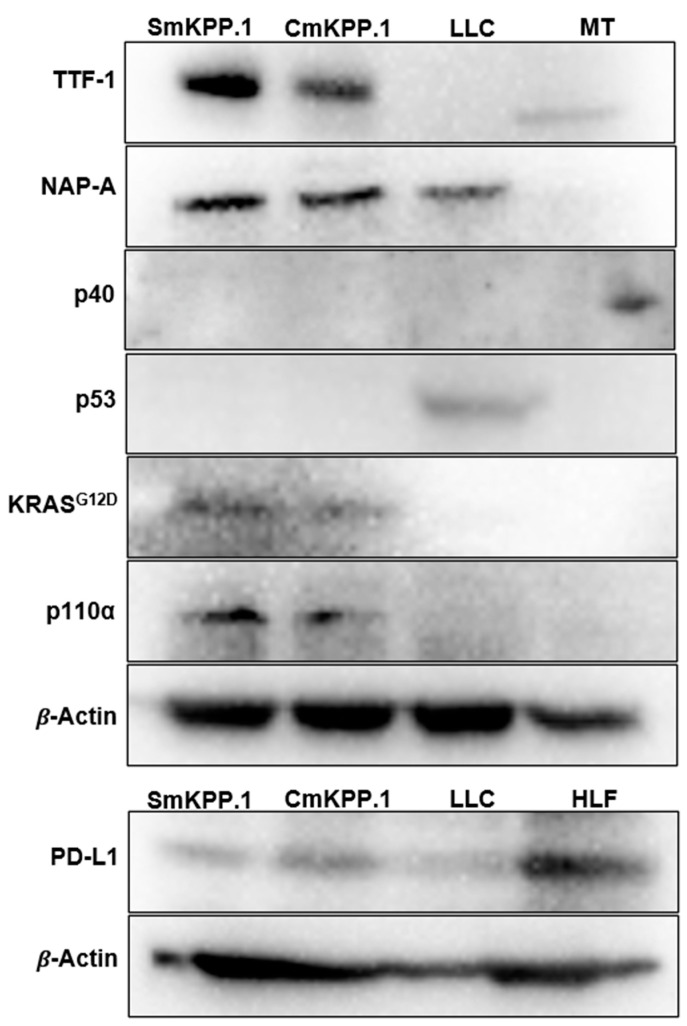
**Western blot of monoclonal cell lines SmKPP.1, CmKPP.1 and LLC.** Both SmKPP.1 and CmKPP.1 exhibit typical tumor markers of human lung adenocarcinoma including the presence of proteins TTF-1 (40 kDA), NapA (35 kDA), and absence of p40 (40 kDA); they also demonstrate the absence of gene product p53 (53 kDA), and the presence of KRASG12D (25 kDA) and p110α (110 kDA). This is in comparison to LLC which is positive for NapA but negative for TTF-1 and p40, positive for p53, and negative for KRASG12D and p110α. PD-L1 is more strongly present in CmKPP.1 compared to SmKPP.1 and LLC. Murine testes (MT) serve as a positive control for p40, and human lung fibroblasts (HLF) serve as a positive control for PD-L1. Cell passage 22 used for SmKPP.1 and passage 16 for CmKPP.1.

We assessed gene products (Figure 2) in SmKPP.1 and CmKPP.1 and included the LLC cell line for comparison. Western blots of cell lysates of both SmKPP.1 and CmKPP.1 confirmed the presence of KRAS^G12D^, overexpression of p110α, and deletion of p53. In contrast, LLC did not express KRAS^G12D^ or p110α, but did express p53. Murine testes (MT) from a C57BL/6 mouse does not express p53 under normal conditions [65] and served as a negative control. Human Lung Fibroblasts (HLF) is known to express PD-L1 [66] and served as a positive control.

Protein expression (Figure 2) specific to lung adenocarcinoma was demonstrated by both SmKPP.1 and CmKPP.1. Both express TTF-1 and NapA; in contrast, LLC only expresses NapA. The absence of tumor protein 40 (p40) was also observed by SmKPP.1 and CmKPP.1, as well as LLC; MT which does express p40, served as a positive control. PD-L1 was more strongly expressed by CmKPP.1 compared to SmKPP.1 and LLC. Thus, protein expression of SmKPP.1 and CmKPP.1 is not only consistent with the majority of human lung adenocarcinoma, but the presence of PD-L1 is predictive of response to immunotherapy.

**Figure 3:**
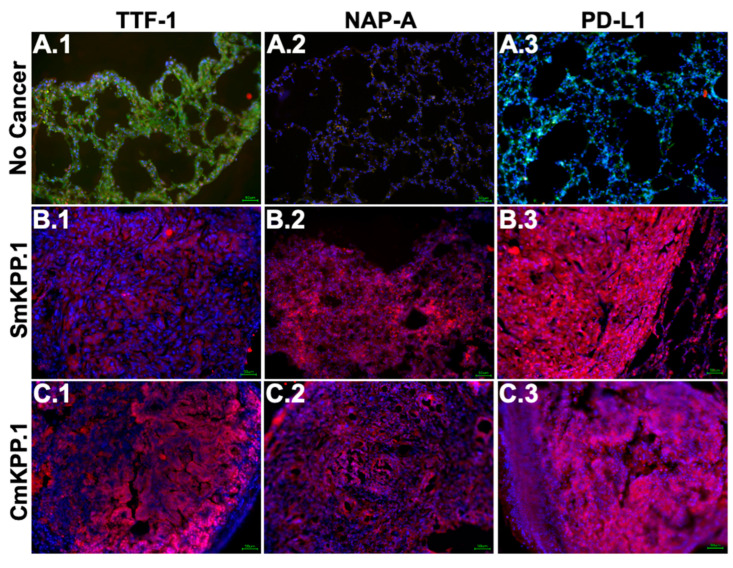
**Immunofluorescence staining of normal, healthy murine lung tissue and orthotopic tumors generated with monoclonal SmKPP.1 or CmKPP.1 cells**. Images demonstrate that tumors generated by SmKPP.1 are positive for TTF-1 ((**B.1**), red), NapA ((**B.2**), red), and PD-L1 ((**B.3**), red); tumors generated by CmKPP.1 are also positive for TTF-1 ((**C.1**), red), NapA ((**C.2**), red), and PD-L1 ((**C.3**), red). Lung tissue from normal control mice without tumor exhibits a low expression of TTF-1 ((**A.1**), arrows), and the absence of NapA (**A.2**) and PD-L1 (**A.3**). All tissue slices were stained for CD31 (green, for endothelial cells, not included in the overlay for SmKPP.1 or CmKPP.1) to provide architecture and counterstained with DAPI (blue, for cellular nuclei). The scale bar represents 50 μm.

### 3.4. In Vivo Tumor Response to Immune Checkpoint Inhibitors (ICI) Simulates Clinical Outcomes

**Figure 4:**
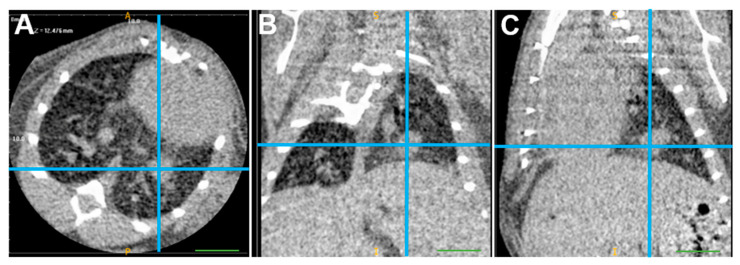
**µCT scan of starting tumor volume of a mouse with an orthotopic tumor generated by 125,000 SmKPP.1 cells.** In this representative mouse, one week after injection of SmKPP.1 cells, the left lung tumor is clearly identified (blue crosshairs) in the axial (**A**), coronal (**B**), and sagittal (**C**) planes using ITK-SNAP software, version 3.8.0. Diameters measured in each plane were then applied to a formula for the volume of an oval, the volume of this orthotopic tumor is 6.19 mm^3^. The scale bar represents 1.0 mm.

**Figure 5:**
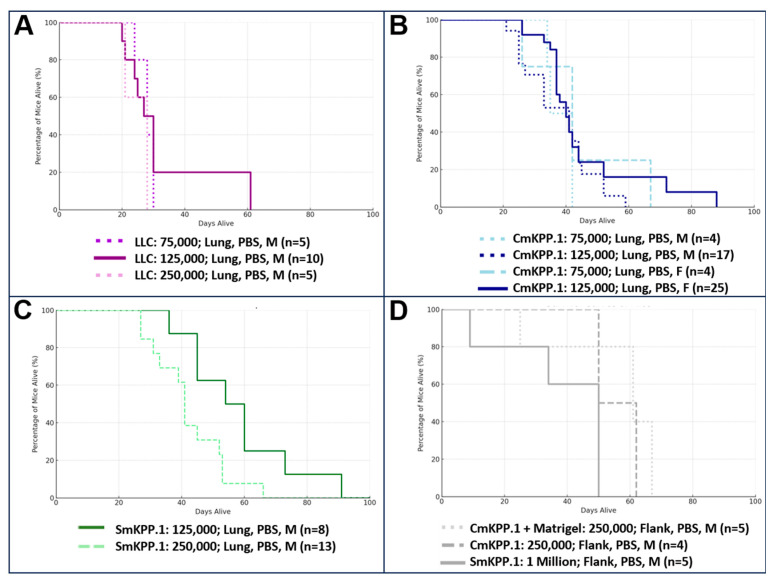
**Different concentrations of LLC, CmKPP.1 or SmKPP.1, have different survival curves.** (**A**) When LLC is injected at a concentration of 75,000, 125,000, or 250,000 there is no difference in survival. (**B**) In addition, when CmKPP.1 is injected at a concentration 75,000 or 125,000 there is no difference in survival. (**C**) However, when SmKPP.1 is injected at a concentration of 125,000 survival is statistically longer compared to a concentration of 250,000, *p* = 0.027. (**D**) Interestingly, when CmKPP.1 at 250,000 or SmKPP.1 at 1 million was injected into the flank, there was no difference in survival.

Ninety-six mice were injected with clone cells, of which n = 18 were treated with immunotherapy. First, we assessed survival when injected with different concentrations of cells into the left lung and microcomputed tomography (µCT) scans (Figure 4) were generally performed one week after orthotopic injection to confirm the presence of intrapulmonary tumors in mice. We compared the survival conditions of male and female mice injected with varying concentrations of LLC, CmKPP.1, or SmKPP.1 into the left lung, and report them in Figure 5. For LLC (Figure 5A), when comparing mice injected with 75,000 clone cells (males, n = 5), 125,000 (males, n = 10), or 250,000 (males, n = 5) there was no difference in survival (Kruskal–Wallis H-test, H Statistic: 1.494, *p*-value: 0.474). For CmKPP.1 (Figure 5B), when comparing mice injected with 75,000 clone cells (males, n = 4), 125,000 (males, n = 17), 75,000 (females, n = 4) or 125,000 (females, n = 25), there was also no difference in survival (Kruskal–Wallis H-test, H Statistic: 1.659, *p*-value: 0.646). However, for SmKPP.1, when comparing mice injected with 125,000 (males, n = 8) and 250,000 (males, n = 13), there is a difference in survival (Mann–Whitney U test, U-statistic: 83; *p*-value 0.027). Observationally, LLC is extremely aggressive; however, injecting mice with lower than 75,000 resulted in inconsistent lung tumor initiation. CmKPP.1 demonstrated no difference between males and females, however, injecting mice with lower than 125,000K resulted in inconsistent lung tumor initiation. SmKPP.1 (Figure 5C), demonstrated the most variance; in males, tumors could be initiated with 125,000 cells, but in females, tumors could not be initiated at this concentration; in both males and females, injecting SmKPP.1 at a concentration of 250,000 was more consistent. When we injected 250,000 CmKPP.1 clone cells into the flank with or without Matrigel (Figure 5D), compared to 1 million SmKPP.1 clone cells, there was no difference in survival (Kruskal–Wallis H-test, H Statistic: 4.595, *p*-value: 0.101). While measuring tumor size with calipers was easier and required less training, we found that mice frequently ate each other’s tumors making it difficult to track tumor progression. Also, it was not uncommon for the flank tumor site to became infected, making it difficult to interpret flow cytometry or effect of immunotherapy. Therefore, we focused only on studying lung cancer in the lung.

**Figure 6:**
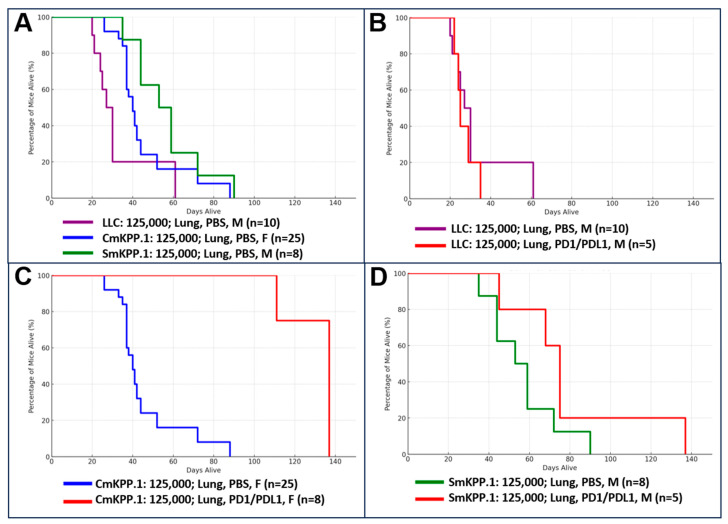
**Survival curves for three cohorts of mice with orthotopic tumors generated by LLC, CmKPP.1 or SmKPP.1, comparing treatment with immunotherapy to control (1XPBS).** Within each cohort, mice underwent weekly intraperitoneal injections of immunotherapy with anti-PD-1 on Tuesdays and anti-PD-L1 on Thursdays; control mice were injected with 1× PBS on the same days. (**A**) Survival of three control cohorts demonstrates that LLC is more aggressive than CmKPP.1 and SmKPP.1; and CmKPP.1 is more aggressive than SmKPP.1. (**B**) Survival of LLC cohort comparing immunotherapy to PBS is consistent with other data that orthotopic tumors generated by LLC most likely has primary resistance to immunotherapy. (**C**) CmKPP.1 cohort and (**D**) SmKPP.1 cohort demonstrate orthotopic tumors are initially responsive to immunotherapy when compared to PBS, but are lethal, suggesting mice develop secondary resistance.

Next, we evaluated response to immunotherapy in three cohorts of mice bearing orthotopic tumors generated from 125,000 cells of LLC (males, n = 15), CmKPP.1 (females, n = 33), or SmKPP.1 (males, n = 13); please note that the mice reported in Figure 6 as “controls” are also included in Figure 5. Summary statistics (mean, standard deviation) with “days alive after injection” as the outcome are as follows: (1) LLC- Control (n = 10): 33, 15.23; Immunotherapy (n = 5): 27, 5.15, (2) CmKPP.1- Control (n = 15): 45.5, 16.77; Immunotherapy (n = 8): 130.5, 12.04, (3) SmKPP.1- Control (n = 8): 57, 17.57; Immunotherapy (n = 5): 80, 34.16. Figure 6A is survival curve of mice from all three cohorts treated with PBS (control), and a Kruskal–Wallis H-test was conducted with days alive after injection as the outcome and there was a difference in survival (H statistic: 11.71; *p*-value: 0.0029). A Mann–Whitney U test between two groups demonstrated a difference between LLC and CmKPP.1 (U statistic: 50, *p*-value: 0.0063), LLC and SmKPP.1 (U statistic: 12, *p*-value: 0.0142) and CmKPP.1 and SmKPP.1 (U statistic: 50.5, *p*-value 0.0386).

Figure 6B is a survival curve of mice in the LLC cohort, comparing mice treated with immunotherapy to mice treated with PBS. Further analyses using a Mann–Whitney U test was conducted with days alive after injection as the outcome and there was no difference in survival (U statistic: 29.0, *p*-value: 0.666). Figure 6C is a survival curve of mice in the CmKPP.1 cohort again comparing mice treated with immunotherapy to control; here a Mann–Whitney U test demonstrated immunotherapy resulted in a statistically significant increase in survival (U statistic: 0, *p*-value: 2.58 × 10^−5^). Lastly, in Figure 6D, the SmKPP.1 cohort demonstrated no difference in survival when treated with immunotherapy compared to control (U-statistic: 9.0, *p*-value: 0.123). A Kruskal–Wallis H-test comparing all three cohorts when treated with immunotherapy demonstrated that there is a difference (H-statistic: 13.65, *p*-value: 0.00109). A Mann–Whitney U test between two groups demonstrated a difference between LLC/immunotherapy vs. CmKPP.1/immunotherapy (U statistic: 0, *p*-value: 0.00264), LLC/immunotherapy vs. SmKPP.1/immunotherapy (U statistic: 0, *p*-value: 0.01193), and CmKPP.1/immunotherapy vs. SmKPP.1/immunotherapy (U statistic 35, *p*-value: 0.02061).

Across all three cohorts, as tumor size increased, mice developed cachexia slowly over time, with progressive weight loss each week. In addition to weight loss, mice were observed to have poor grooming, respiratory distress and an increased work of breathing prior to necropsy, although some mice did not exhibit these signs prior to death. Necropsy findings of all the euthanized mice implicated significant tumor burden as the cause of death. Maximum tumor volume was the entirety of the left hemithorax, with obliteration of normal lung parenchyma and metastasis to the contralateral right lung. None of the mice died of gross hemorrhage, although notably, several were anemic, and upon necropsy, two had pulmonary hemorrhage (notably, both were LLC control mice).

Although our study was not designed to detect survival differences of less than three weeks, collectively these results demonstrate the potential efficacy of immunotherapy slowing tumor progression of CmKPP.1 and SmKPP.1 orthotopic tumors and relative inefficacy for slowing tumor progression of LLC orthotopic tumors. Notwithstanding prolonged survival, it is essential to acknowledge that all mice ultimately succumbed to malignancy.

## 4. Discussion

We have successfully developed and characterized two new, monoclonal, murine lung cancer cell lines, SmKPP.1 and CmKPP.1, and used them to generate syngeneic, orthotopic murine models of lung adenocarcinoma. We demonstrated that both cell lines closely resemble human lung adenocarcinoma based on typical histology, protein expression patterns, genetic markers, and immunogenic antigen expression frequently used by pathologists and clinicians in the diagnosis and management of human NSCLC subtypes [19,20]. In addition, we used immunofluorescence to demonstrate how orthotopic tumors derived from SmKPP.1 or CmKPP.1 have distinct patterns of TTF-1, NapA, and PD-L1 protein localization. Lastly, we demonstrated that mice with orthotopic SmKPP.1 or CmKPP.1 tumors, when treated with immunotherapy, survive longer than control mice, and longer than mice with orthotopic LLC tumors treated with immunotherapy. Yet, despite the observed increase in survival time, lethality remained 100% in both cohorts of mice with SmKPP.1 or CmKPP.1 tumors, and necropsy findings of all mice demonstrated significant tumor burden and metastasis, suggesting secondary resistance.

It is estimated that in the US, there will be over 234,000 new cases of lung and bronchus cancers in 2024 [67], with ~40% (~94,000 new cases) being lung adenocarcinoma. Our robust autochthonous (genetic) model mimics the progression of human lung adenocarcinoma and builds on the seminal work of Dr. Nemenoff, who characterized the immunogenic orthotopic lung cancer model [49,51,68]. According to The Cancer Genome Atlas (TCGA), the three main KRAS mutations of lung adenocarcinoma [69,70] are G12C (10–13%), G12V (5–6%) and G12D (4–5%). While the G12C and G12V mutations are most prevalent in current and former smokers, the G12D mutation is the most prevalent in never smokers [71]. Therefore, our model with the G12D mutation complements existing models with the G12C mutation (CMT54, CMT55, mKRC.1 [72] and LLC) or the G12V mutation (CMT167 [72]). Furthermore, since 53% of lung adenocarcinoma have a mutation in the TP53 gene [73], our model with a deleted p53 complements other models with a p53 mutation (CMT54 and CMT55) or models that express wild-type TP53 (CMT 167 and mKRC.1 [72]). Lastly, with 25–30% of lung adenocarcinoma exhibiting a mutation within the PI3K-AKT-mTOR pathway [74,75], our model with a myristoylated p110α complements models with other metabolic mutations.

The protein expression profiles, and corresponding histologic phenotypes of SmKPP.1 and CmKPP.1 represent major strengths of our cell lines compared to existing murine lines. Specifically, our cells are TTF-1 and NapA positive and p40 negative, and when used to generate orthotopic tumors, exhibit histology typical of solid human lung adenocarcinoma. CMT167 cells are TTF-1 positive [76] and exhibit histology consistent with lung adenocarcinoma but appear more representative of the papillary sub-type rather than solid subtype [5]. This histologic phenotype could explain the high metastatic potential of CMT167, but because papillary adenocarcinoma represents 7–12% of all human lung adenocarcinoma [35,77,78] this subtype may be less representative of clinical lung cancer than solid adenocarcinoma subtypes. LLC cells, by contrast, are TTF-1 negative [76,79,80], NapA positive, p40 negative, and histologic phenotype is conflicting [81,82]. Orthotopic tumors generated by LLC cells were initially described as having non-squamous histology, resembling human bronchoalveolar carcinoma rather than classical adenocarcinoma [81]; later reports suggested the presence of squamous features, e.g., flat and elongated morphology [82]. Finally, while protein expression profiles have not been published for MAD109 or KLN-205 cell lines, initial histological descriptions was more consistent with squamous cell carcinoma in that microvilli project from the cell surface and tight intercellular bridges connect adjacent cells; however later they were described to exhibit features of adenocarcinoma, e.g., large and round morphologies [83].

The gene products of SmKPP.1 and CmKPP.1 are consistent with the original GEM genotypes: they lack p53, demonstrate upregulation of the KRAS^G12D^ protein, and myristoylation of p110α. CMT167 has an endogenous KRAS^G12V^ mutation and presence of p53 [84]; recently it underwent editing to have a KRAS^G12C^ mutation [72]. LLC has the more common, KRAS^G12C^ mutation [72], the presence of p53 [76] and the PI3K pathway is inactive, demonstrated by the absence of p-AKT [76]. To the best of our knowledge, this data is not known for cell lines MAD109 or KLN-205. The clinical significance of specific KRAS mutations is that they have different prognostic implications [85] and activate different downstream signaling pathways [85,86,87]. Furthermore, the presence of p53 mutations has a cooperative effect on KRAS mutations [88,89] and PI3K pathway mutations in adenocarcinoma can coexist with KRAS mutations [90] and are commonly associated with tyrosine kinase inhibitor and immune checkpoint inhibitor resistance [40,41]. Currently, KRAS targeted treatments are clinically available for G12C specific mutations but are ineffective against G12V or G12D specific mutations. As there are several investigational drugs that target G12D specific mutations [91,92,93,94,95,96], p53 mutations [91], and PI3K inhibitors [97,98,99], our clone cells could be used to evaluate the efficacy of these investigational drugs, both alone and in combination with immunotherapy, as well as in studying mechanisms of resistance.

Clinically, patients who benefit from immune checkpoint inhibitors (ICI) are categorized as responders, constituting approximately 15% to 25% [47,48,100] of lung adenocarcinoma patients, and response to anti-cancer drugs such as ICIs is frequently reported as either or both, progression free survival (PFS) and/or overall survival (OS). However, the International Association for the Study of Lung Cancer Classification recently placed greater emphasis on OS, due to the lack of correlation between tumor diameters (in the largest dimension) and any meaningful clinical outcome, including survival. With that context and since we could not know how orthotopic tumors of our lines would respond to immunotherapy from the start, initially we chose to measure therapeutic response as overall survival (OS) alone. Thus, after comparing ICI-treated mice with orthotopic tumors of our lines to those with orthotopic tumors of other lines, we feel confident making several significant inferences about our orthotopic tumor progression and resistance patterns: First, because orthotopic tumors generated with either SmKPP.1 or CmKPP.1 demonstrated an increased OS compared to LLC, orthotopic SmKPP.1 or CmKPP.1 tumors are not likely to exhibit primary resistance to ICIs. Second, unlike orthotopic tumors generated with CMT167, which demonstrate a remarkable response to immunotherapy in prior trials [49], mice with SmKPP.1 or CmKPP.1 all died from tumor burden and are thus likely to have developed secondary resistance to ICIs. Overall, this behavior in response to ICI suggests mice with orthotopic tumors of SmKPP.1 or CmKPP.1 behave in a manner closely resembling clinical responders.

Accordingly, subtle differences in the response to immunotherapy between the cell lines are worth noting. While mice with CmKPP.1 tumors had a statistically significant prolonged survival when treated with immunotherapy compared to mice with SmKPP.1 tumors, this may be due to the lack of power in the SmKPP.1 cohort. In addition, further optimization of the concentration of SmKPP.1 in males and females is currently underway and may also be a reason for the difference in statistical significance. Considering that the oncogenic driver mutations are identical between our two cell lines, and that TPS is effectively 100% in both models due to the presumed monoclonal nature of the lines (confirmed by IF), it is possible that other genetic differences are responsible. Further genetic testing is ongoing. Additionally, other factors such as doubling times and metabolic characteristics of the two different tumors may also play a role in their susceptibility to treatment and the subsequent development of resistance. To better comprehend underlying mechanisms driving a differential response to immunotherapy, further investigations are warranted into the interaction between our oncogenic drivers and tumor protein expression markers (e.g., TTF-1, NapA, PD-L1) to improve patient outcomes.

## 5. Limitations

Our study has several limitations that should be acknowledged. Most importantly, we have not completed full characterization of female mice for these experiments. The fact that both S**m**KPP.1 and C**m**KPP.1 were derived from **male** mice was inadvertent (see Methods) as they were part of a survival experiment where Cre viruses were used to initiate cancer and male mice happen to meet our tumor volume criteria for clone cell generation. Interestingly, for CmKPP.1, there was no difference in male vs. female mice; however, current ongoing investigation suggests this may not be the case for SmKPP.1. In the future, we will report characterization of these cell lines derived from male mice in female mice, and we will develop cancer cell lines from female mice.

Next, we did not conduct formal genetic testing of SmKPP.1 and CmKPP.1 cell lines, or of orthotopic tumors generated by the new cell lines, and we did not investigate the tumor microenvironment. While each cell line started as a single cell and most likely remains genetically homogeneous, there is a possibility that genetic drift occurred during cell line passages. Additionally, after injection of either cell line, genetic heterogeneity can develop in vivo, in response to the tumor microenvironment. Therefore, genetic heterogeneity may be present, and together with tumor microenvironmental differences, could account for variable responses to immunotherapy. For example, the tumor microenvironment was implicated in determining the efficacy of immunotherapy when administered to mice with orthotopic tumors of CMT167 cells [49]. In the future, genetic testing will be conducted on earlier and later passages of SmKPP.1 and CmKPP.1 cells to determine whether they are homogeneous or heterogeneous; investigation of the tumor microenvironmental is currently ongoing.

Finally, the absence of reporting response to immunotherapy as a function of tumor volumes is a limitation that prevents us from comparing between labs and different treatments. When we were optimizing the method to investigate this mouse model of lung cancer, to account for variability in tumor initiation, we eventually moved to using µCT scans to determine a mouse’s starting tumor volume before administering immunotherapy or control. Therefore, we did not obtain serial µCT scans on all mice, primarily due to changes in protocol, but also due to practical concerns. One practical concern is that radiation dose is a confounding factor, and we have not yet optimized whether to fix the number of scans or fix the scanning interval. If we fixed the number of scans (e.g., four total scans per mouse), we need to know when each mouse will die, which means we would need to generally know if the tumor is progressing, regressing, or lacks progression. If we fixed the number of scans (e.g., weekly), some mice will undergo significantly more scans if they survive longer, whereby there is risk that the radiation affects tumor progression or increases the risk of pneumonitis. Determining the optimal schedule of µCT scans is currently underway. A second practical concern is that manual tumor volume segmentation is time- and cost-prohibitive for small labs; currently we are working on a semi-automated algorithm.

## 6. Conclusions

We developed, authenticated, and clinically characterized two new immortal, monoclonal, murine lung adenocarcinoma cell lines, SmKPP.1 and CmKPP.1, which can be used to generate orthotopic tumors in immunocompetent C57BL/6 mice. We also demonstrated an improvement in the survival of mice with orthotopic tumors derived from our cell lines when treated with immunotherapy, compared to LLC, which displays primary resistance to immunotherapy. Yet, we showed how orthotopic tumors of our cells are lethal despite an initial response to immunotherapy. Thus, our cell lines exhibit several distinct advantages over existing cell lines, particularly with respect to the investigation of tumor-immune interactions, as well as mechanisms and pathways of secondary resistance to immunotherapy. From the inception of our project, our primary objective has been to improve survival in patients with advanced NSCLC. Now, with an orthotopic model that exhibits similar architecture, gene products, and behavior as human lung adenocarcinoma, we offer scientists a complimentary model to further bridge the gap between scientific investigation and clinical practice.

## Data Availability

The data that support the findings of this study are available from the corresponding author on reasonable request.
